# Current view on the functional regulation of the neuronal K^+^-Cl^−^ cotransporter KCC2

**DOI:** 10.3389/fncel.2014.00027

**Published:** 2014-02-06

**Authors:** Igor Medina, Perrine Friedel, Claudio Rivera, Kristopher T. Kahle, Nazim Kourdougli, Pavel Uvarov, Christophe Pellegrino

**Affiliations:** ^1^INSERM, Institut de Neurobiologie de la Méditerranée (INMED)Marseille, France; ^2^Aix-Marseille Université, UMR901Marseille, France; ^3^Neuroscience Center, University of HelsinkiHelsinki, Finland; ^4^Department of Cardiology, Manton Center for Orphan Disease Research, Howard Hughes Medical Institute, Boston Children's HospitalBoston, MA, USA; ^5^Department of Neurosurgery, Massachusetts General Hospital and Harvard Medical SchoolBoston, MA, USA; ^6^Institute of Biomedicine, Anatomy, University of HelsinkiHelsinki, Finland

**Keywords:** KCC2, intracellular chloride, GABA, neurons

## Abstract

In the mammalian central nervous system (CNS), the inhibitory strength of chloride (Cl^−^)-permeable GABA_A_ and glycine receptors (GABA_A_R and GlyR) depends on the intracellular Cl^−^ concentration ([Cl^−^]_i_). Lowering [Cl^−^]_i_ enhances inhibition, whereas raising [Cl^−^]_i_ facilitates neuronal activity. A neuron's basal level of [Cl^−^]_i_, as well as its Cl^−^ extrusion capacity, is critically dependent on the activity of the electroneutral K^+^-Cl^−^ cotransporter KCC2, a member of the *SLC12* cation-Cl^−^ cotransporter (CCC) family. KCC2 deficiency compromises neuronal migration, formation and the maturation of GABAergic and glutamatergic synaptic connections, and results in network hyperexcitability and seizure activity. Several neurological disorders including multiple epilepsy subtypes, neuropathic pain, and schizophrenia, as well as various insults such as trauma and ischemia, are associated with significant decreases in the Cl^−^ extrusion capacity of KCC2 that result in increases of [Cl^−^]_i_ and the subsequent hyperexcitability of neuronal networks. Accordingly, identifying the key upstream molecular mediators governing the functional regulation of KCC2, and modifying these signaling pathways with small molecules, might constitute a novel neurotherapeutic strategy for multiple diseases. Here, we discuss recent advances in the understanding of the mechanisms regulating KCC2 activity, and of the role these mechanisms play in neuronal Cl^−^ homeostasis and GABAergic neurotransmission. As KCC2 mediates electroneutral transport, the experimental recording of its activity constitutes an important research challenge; we therefore also, provide an overview of the different methodological approaches utilized to monitor function of KCC2 in both physiological and pathological conditions.

## Introduction

Since the discovery of the dichotomic action of GABAergic neurotransmission more than two decades ago (Mueller et al., 1984; Ben-Ari et al., 1989), important progress has been made in the understanding of the role of intraneuronal Cl^−^ concentration ([Cl^−^]_i_) in determining the action of GABA and glycine on post-synaptic neurons. It is now clear that [Cl^−^]_i_ is critical for proper functioning of the CNS in physiological conditions, and alterations of [Cl^−^]_i_ are observed in multiple neurological disorders that are characterized by GABAergic disinhibition (reviewed by Ben-Ari et al., 2007; Kahle et al., 2008)

One of the key molecules responsible for determining [Cl^−^]_i_ in mature mammalian neurons, and also impaired in numerous pathological conditions resulting in deranged [Cl^−^]_i_ homeostasis, is the K^+^-Cl^−^ cotransporter KCC2, a neuron-specific Cl^−^ extruder of the cation-Cl^−^ cotransporter gene family *SLC12*. KCC2 plays a critical role in setting neuronal [Cl^−^]_i_; its deficiency (Rivera et al., 1999) or absence (Hübner et al., 2001; Woo et al., 2002; Balakrishnan et al., 2003; Zhu et al., 2005) raises [Cl^−^]_i_ and thereby decreases the inhibitory strength of GABA and glycine, whose cognate receptors are ligand-gated ion channels permeable to Cl^−^ and HCO^−^_3_ ions. Impaired KCC2 activity and consequent increases of [Cl^−^]_i_ have been documented in epilepsy (Palma et al., 2006; Huberfeld et al., 2007), neuropathic pain (Coull et al., 2003), and spasticity following spinal cord injury (Cramer et al., 2008; Shulga et al., 2008; Boulenguez et al., 2010). These and other works (see Kahle et al., 2008; Blaesse et al., 2009; Ben-Ari et al., 2012 for detailed reviews) have suggested that decreased KCC2 activity is a principal cause of the rise in [Cl^−^]_i_, that leads to the depolarizing action and enhancement of neuronal network activity in these paradigms.

Recent findings have provided novel insights into the dynamics of KCC2 activity and [Cl^−^]_i_ homeostasis in different pathology-related experimental conditions, revealing the existence of complex responses depending on the type of neuronal activity and the timing of observation (e.g., Khirug et al., 2010; Pellegrino et al., 2011; Shulga et al., 2012). In parallel, other studies strongly contributed to the understanding of the signaling pathways controlling KCC2 activity (e.g., Puskarjov et al., 2012; Bos et al., 2013; Gagnon et al., 2013; Ivakine et al., 2013; Markkanen et al., 2013; De Los Heros et al., 2014). In the present work we review recent compelling findings, highlight unresolved questions, and discuss the technical challenges that underlie the study of KCC2.

## Overview of KCC2 basic properties

Several recent reviews have provided detailed analysis of the KCC2, its expression profile, physiological roles and implications in different neurological disorders (e.g., Gamba, 2005; Medina and Chudotvorova, 2006; Kahle et al., 2008, 2013; Blaesse et al., 2009; Chamma et al., 2012; Gagnon and Delpire, 2013). Here, to avoid repeating these reviews, we highlight briefly only some of KCC2 properties required as background for further discussion on the molecular determinants of the activity-dependent control of the transporter.

KCC2 is one of nine cation-chloride cotransporters (CCCs) encoded by *SLC12* family genes. It shares high homology with three other K^+^-Cl^−^ cotransporters (KCC1, KCC3, and KCC4), however it is distinct from these and other CCCs transporters by its unique importance for functioning of CNS: (i) KCC2 is the only CCC transporter expressed preferentially in neurons; (ii) it is characterized by a progressive increase in expression during development; (iii) KCC2 shows activity in isotonic conditions; (iv) KCC2 plays a critical role in establishing neuronal [Cl^−^]_i_ and (v) it is involved in the control of numerous neuronal processes including migration, dendritic outgrowth, formation of synaptic connections, and spine morphology.

KCC2 was cloned in 1996 by homology with the KCC1 cotransporter from a rat brain cDNA library (Payne et al., 1996). This, and numerous other studies have characterized KCC2 as a transporter expressed exclusively in neurons of the CNS (reviewed by Blaesse et al., 2009), however some recent works showed also KCC2 expression in human fetal lens epithelial cell line (Lauf et al., 2008, 2012), chicken cardiomyocytes (Antrobus et al., 2012) and cancer cells (Wei et al., 2011) suggesting that functioning of KCC2 extends beyond the CNS. The silencing of KCC2 in mice (Hübner et al., 2001; Woo et al., 2002; Zhu et al., 2008; Khalilov et al., 2011) as well as KCC2-like genes in worms (CE-KCC-2, Tanis et al., 2009; Bellemer et al., 2011) and flies (dm-*kcc*, Hekmat-Scafe et al., 2006, 2010) produces severe changes of the neuronal network properties leading to appearance of seizure activity, increased neuron hyperexcitability and modifyied formation of synaptic connections. The silencing of other members of *SLC12* gene family expressed in brain (NKCC1, KCC1, KCC3, and KCC4) lead to much less pronounced changes of the phenotype (see Gagnon and Delpire, 2013 for most recent review on genetically engineered CCC knockouts).

**Figure 1:**
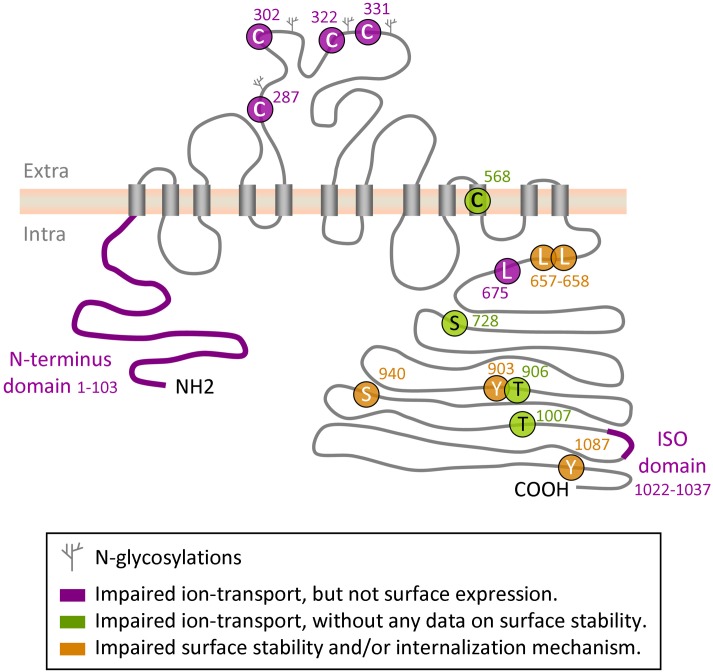
**Regulatory sites on KCC2 protein**. Depicted is a schematic representation of the amino acid residues and domains in KCC2 that have been implicated in the control of intrinsic KCC2 transport activity or in its cell surface stability and/or membrane internalization.

The secondary and tertiary structure of KCC2 and the other KCCs is unknown. Based on hydropathy analysis of KCC2 protein, Payne et al. (1996) proposed a putative model of KCC2, including 12 transmembrane domains and intracellular N- and C-termini. The authors predicted four glycosylation sites on the 3^rd^ putative extracellular loop and highlighted a potential tyrosine phosphorylation site at position 1087. So far, this is the only model proposed. It is widely explored in drawings illustrating putative structure of the KCC2 in many reviews and original data publications (e.g., Li et al., 2007; Zhao et al., 2008). The KCC2 scheme drawn in the present review (Figure 1) has been also inspired by the original drawing by Payne et al. (1996). The functional importance of different structural elements of the KCC2 is also little studied. The region forming ion-transporting element of the KCC2 and mechanism controlling intrinsic activity of the transporter remain unclear (see below for more details). An important aspect of KCC2 that may affect activity of this transporter is its ability to oligomerize, and this issue may be one of the most challenging in the elucidation of KCC2 function and regulation.

KCC2 is a secondary-active electroneutral transporter that carries K^+^ and Cl^−^ ions in a 1:1 stoichiometry. The direction of the ion transport depends on the sum of chemical gradients of K^+^ and Cl^−^ (Payne, 1997). When close to the chemical equilibrium, even slight changes in the concentration of either ion lead to a change of the direction of the ion transport (Payne, 1997). This property of the KCC2 is largely used during measurements of the KCC2 activity by mean of ^86^Rb^+^, Tl^+^ or NH^+^_4_ influx (see inset Box for details).

Box 1 **How to Measure KCC2 Ion-Transport Activity.**

Due to its electroneutrality, the KCC2 transport does not generate ion current. Therefore the electrophysiological approaches used to directly monitor opening of ion channels are not applicable to the analysis of KCC2, as well as of other members of CCC family. To bypass this problem, researchers measure consequences of transporter activity, i.e., the dynamic changes of the intracellular concentration of either K^+^ or Cl^−^.

***Flame photometry***. The first studies characterizing activity of the endogenous cation-Cl^−^-dependent ion transporters determined changes in the intracellular content of Na^+^, K^+^, and Cl^−^ by flame photometry (Geck et al., 1980) or by analysing ^24^Na^+^ and ^40^K^+^ radioactivity (Kregenow, 1971). Amazingly, these and other pioneer works studying ion fluxes through endogenous transporters determined the exact stoichiometry of Na^+^-K^+^-Cl^−^ (1:1:2) and K^+^-Cl^−^ (1:1) co-transport, characterized the ion specificity, and described the requirement of Cl^−^ for operation of Na^+^-K^+^-Cl^−^ and K^+^-Cl^−^ transporters (Haas, 1994). These properties were confirmed many times and are considered presently as landmarks for the NKCC and KCC subfamilies of transporters (Blaesse et al., 2009).

***86RB^+^ flux assay***. A more modern approach to analyse the NKCC and KCC activity consists in measurement of the ^86^Rb^+^ flux through the membrane. This approach was used successfully to clone and characterize NKCC1 (Gamba et al., 1994; Xu et al., 1994), KCC1 (Gillen et al., 1996) and KCC2 (Payne, 1997), and continues to be extensively utilized for characterization of different mutants and variants of CCC.

Although the above approaches strongly contributed to study the functional properties of CCC in homogenous cell preparations (erythrocytes, oocytes, different heterologous cell lines), they have limited applications for studying tissues composed of different cell types (i.e., brain slices, neuronal cultures). For example, using ^86^Rb^+^ it is difficult to discriminate in brain slices between the activity of NKCC1 in neuronal cells and surrounding glial cells. Moreover, the radioactive approach does not allow resolution of ion concentration changes at the subcellular level. Finally, the use of radioactive isotope raises the questions of potential toxicity and health hazard and imposes important security restriction.

***TL^+^ flux assay***. This method is based on the use of Thallium (Tl^+^) as the K^+^ tracer. Recent reports have shown that Tl^+^ binds with high affinity to the K^+^ ion site on the KCC2 (Zhang et al., 2010) and NKCC2 (Carmosino et al., 2013) and, once having crossed the plasma membrane, could be efficiently detected using metal-sensitive fluorescent dyes (Delpire et al., 2009; Hartmann et al., 2010; Zhang et al., 2010; Carmosino et al., 2013).

***NH_4_*^+^ flux assay**. NH^+^_4_ uptake measures indirectly the activity of KCCs and NKCCs, which are highly-efficient carriers of NH^+^_4_ at their K^+^ site (see Williams and Payne, 2004 for detailed review and analysis of KCC2 selectivity). Several groups have used this property of CCC proteins to estimate KCC2 activity by measuring NH^+^_4_-dependent changes of pH_i_ (Williams and Payne, 2004; Titz et al., 2006; Hershfinkel et al., 2009). This approach allows the analysis of KCC2 activity on the cellular and subcellular level as well as the estimation of KCC2 activity in real time. The inconveniences of the approach are related to the complex changes of the pH_i_ in response to the application of the solution containing NH^+^_4_ (KCC2-independent alkalinisation followed by KCC2-dependent acidification) and possible contamination of pH_i_ changes by functioning of other transporters, channels and ion exchangers. In addition, similarly to Tl^+^ method, the NH^+^_4_ approach measures KCC2 activity in reverse mode that increases [Cl^−^]_i_ and places recorded cells in non-physiological conditions.

Thus, the methods described above allow measurement of the intrinsic transport carried by KCC transporters. These methods are designed to measure cation influx, i.e., to analyse KCC2 activity in reverse mode that is not its physiological function and will cause an increase of [Cl^−^]_i_. Accordingly, the potency of Tl^+^ and NH^+^_4_ methods to detect the ion flux mediated by endogenous KCC transporters under physiological conditions remains to be demonstrated.

A recording of Cl^−^ is a highly efficient alternative to the cation methods of CCC activity measurements under conditions close to physiological. Numerous works have shown that KCC2 activity leads to strong changes in the intracellular Cl^−^ concentration (reviewed by Ben-Ari et al., 2007; Blaesse et al., 2009). The estimate ion of [Cl^−^]_i_ in neuronal cells could be achieved via different methods. Here we provide a brief description of different ways of estimating [Cl^−^]_i_ and emphasize their relative advantages and limitations.

***Gramicidin perforated patch clamp (GPPC) recording***. The method is based on the use of the antibiotic gramicidin that makes cation-selective pores in the membrane; the patch pipette is filled with gramicidin and thus, preserves [Cl^−^]_i_ (Ebihara et al., 1995; Kyrozis and Reichling, 1995). The concentration of Cl^−^ is estimated using the Nernst equation after recording the reversal potential of GABA_A_ or glycine receptor-channels that are Cl^−^-permeable. Using the GPPC approach, multiple studies have characterized KCC2-dependent changes in [Cl^−^]_i_ (Balakrishnan et al., 2003; Chudotvorova et al., 2005; Lee et al., 2005; Nardou et al., 2011; Ivakine et al., 2013). The main advantage to GPPC recording is the estimation of [Cl^−^]_i_ with high precision at single cell level. Unfortunately, this method has numerous limitations that should be taken into consideration: (i) it affects [K^+^]_i_, given that KCC2 uses K^+^ as main co-carrier, this should be taken into consideration when interpreting the result obtained with this method; (ii) most of the measurements using GPPC are made in voltage clamp mode of patch clamp recording, and the imposed holding potential provides an additional driving force for Cl^−^ ions, affecting the kinetics of Cl^−^ influx or efflux, and thus possibly confounding interpretations of [Cl^−^]_i_; (iii) the GPPC method has limited application to record [Cl^−^]_i_ in immature neurons, which harbors high input resistance (Tyzio et al., 2003).

***Single GABA_A_ channel recording (SGCR)***. This method is a non-invasive way to estimate the driving force of GABA_A_ using cell-attached recording of single GABA_A_ receptor currents. This approach was used successfully to characterize the developmental shift of GABA from depolarizing to hyperpolarizing (Tyzio et al., 2006, 2008). The main advantage of this method is that it is a non-invasive way of estimating the exact difference between the levels of the resting membrane potential and the reversal potential of GABA_A_R-mediated currents. Limiting points of the method are that there is a labor intensive aspect to data recording and analysis, and there is a requirement of simultaneous single-channel-based recording of resting membrane potential (Tyzio et al., 2003, 2008).

***Soma-to-dendrite Cl^−^ gradient***. To assess Cl^−^ homeostasis and the dynamics of chloride removal in cortical neurons, Jarolimek et al. (1999) and Kelsch et al. (2001) proposed an elegant approach based on whole cell recording of the differences between E_GABA_ in soma and dendrites. The E_GABA_ in the soma is determined by the high [Cl^−^]_i_ diffused from the patch pipette, whereas E_GABA_ in dendrites is more negative (i.e., [Cl^−^]_i_ is lower due to active extrusion by KCC2) (Kelsch et al., 2001). This approach was successfully used by several groups to monitor KCC2 activity in different types of neurons (Khirug et al., 2005; Li et al., 2007; Chamma et al., 2013) and neuronal compartments (Khirug et al., 2008). The advantage of the approach is that it allows a determination of “pure” Cl^−^-extrusion capacity under given experimental conditions. The inconvenience is that the values could be strongly influenced by efficiency of neuron perfusion with intracellular solution (serial resistance, dendrites diameter). In addition, the cell perfusion with intracellular solution may affect KCC2 phosphorylation and, thus, modify its activity.

***Fluorescent probes (DYES)***. The recent achievements in cell biology and microscopy have promoted the use of different Cl^−^-sensitive fluorescent probes for analysis of neuronal Cl^−^ homeostasis. The first set of markers were Cl^−^-sensitive dyes (quinolinium Cl^−^ indicators) exploiting the property of halides to quench the fluorescence of heterocyclic organic compounds with quaternary nitrogen (Chen et al., 1988; Verkman, 1990). These compounds have relatively good sensitivity and selectivity to Cl^−^, and have been used in a variety of preparations. A disadvantage is that a ratiometric recording cannot be performed and their use is strongly limited due to a strong bleaching and the presence of a significant leakage rate (see review Bregestovski et al., 2009). The use of two-photon microscopy has largely resolved bleaching-related problems, and has caused a rediscovery in the use of the quinolinium Cl^−^ indicators (Kovalchuk and Garaschuk, 2012).

***YFP-based markers***. A more recent and promising method for non-invasive analysis of Cl^−^ is based on the halide-binding properties of yellow fluorescent protein (YFP) and its derivatives (Wachter and Remington, 1999; Jayaraman et al., 2000). Based on Cl^−^-sensitive variants of YFP, ratiometric probes allowing estimation of the approximate level of [Cl^−^]_i_ have been created. The first ratiometric Cl^−^ indicator was designed by fusion of YFP with cyan fluorescent protein (CFP) through a polypeptide linker (Kuner and Augustine, 2000). In this construct, called Clomeleon, CFP served as a reference point for normalizing expression levels. The apparent *EC*_50_ *was* 167 mM. Later a more sensitive probe was developed, termed “Cl-Sensor,” composed of CFP and a mutated form of YFP with higher Cl^−^ sensitivity (YFP_Cl_, the apparent *EC*_50_~ 30–50 mM) (Markova et al., 2008; Waseem et al., 2010). Finally, recent work reported engineering of one more Cl^−^ sensitive ratiometric construct called SuperClomeleon that apparently has five-fold higher Cl^−^ sensitivity than Clomeleon (Grimley et al., 2013). Transgenic mice encoding Clomeleon (Berglund et al., 2006) and Cl-Sensor (Batti et al., 2013) have been successfully created.

Both Clomeleon and Cl-Sensor have been effectively used to characterize changes in [Cl^−^]_i_ driven by KCC2 and/or NKCC1 in neuronal cells (Dzhala et al., 2005; Duebel et al., 2006; Pellegrino et al., 2011; Chamma et al., 2013). These YFP-based Cl^−^-sensitive biosensors have a number of advantages: (i) they show much more stable fluorescence at long-lasting monitoring as compared to fluorescent dyes; (ii) they allow excitation in the visible range of wavelength; (iii) they can be targeted to specific cell types either using plasmid transfection (Waseem et al., 2010; Pellegrino et al., 2011; Chamma et al., 2013) or by transgenic creation of animals with knocked-in genes (Berglund et al., 2006; Batti et al., 2013); (iv) they have a high molecular weight, which prevents the diffusion of the indicators from cells and (v) they are suitable for ratiometric measurements. The limiting points of both Clomeleon and Cl-Sensor are: (i) the relatively high rate of bleaching of the fluorescent signal (Kuner and Augustine, 2000; Berglund et al., 2011), (ii) the sensitivity of the probe to intracellular pH (pH_i_) and some organic anions (Jayaraman et al., 2000).

To avoid bleaching of Clomeleon, several groups have successfully used two-photon microscopy (reviewed by Berglund et al., 2011). We reported an alternative method that allows a stable ratiometric recording of Cl-Sensor fluorescence using a conventional epifluorescent microscope (Friedel et al., 2013). The method is based on the discovery of transient inactivation of Cl-Sensor by short wavelength (440 nm) blue light and the corresponding modification of excitation light pass and fluorescence recording procedure. The bypassing of the problem of pH sensitivity of YFP-derived Cl^−^ markers is more problematic as many cellular processes, including activation of GABA_A_ receptors, often involve concomitant changes in both Cl^−^ and H^+^ ion concentration (Russell and Boron, 1976; Kaila et al., 1989). Therefore, a researcher using YFP-based sensors as Cl- indicators should be aware that, even after precise calibration of the set-up, the obtained values of [Cl^−^]_i_ are only indicative.

***^E2^GFP-based Cl^−^ markers***. To circumvent the problem of the pH_i_ sensitivity, Arosio et al. (2010) developed a new ratiometric biosensor (ClopHensor) composed of the Cl^−^ and pH sensitive GFP mutant, ^E2^GFP, fused to monomeric DsRed fluorescent protein and allowing simultaneous measurement of Cl^−^ and pH. Some new modifications of this sensor allowing its membrane targeting and improving pH sensitivity have also been described (Mukhtarov et al., 2013). Another recent work (Raimondo et al., 2013) developed one more ClopHensor derivative designated for expression into neuronal cells (ClopHensorN). Authors found that, when expressed in neurons, DsRed component of ClopHensor formed dense intracellular aggregations that compromised correct ratiometric measurement. The problem has been resolved by replacing DsRed with a tandem dimer tomato (TdTomato). Raimondo et al. (2013) performed also detailed analysis of the dynamic of neuronal [Cl^−^]_i_ and pH_i_ following activation of synaptic and extrasynaptic GABA_A_ receptors that could serve as manual for future works.

In summary, there is a large number of methodological approaches successfully used to assay indirectly the KCC2 activity. So far, each approach has some advantages, but has also limitations that must be taken into account when interpreting results. This is why many groups use a combination of at least two different approaches to corroborate results (i.e., Williams and Payne, 2004; Li et al., 2007; Hershfinkel et al., 2009; Pellegrino et al., 2011; Chamma et al., 2013).

## Diversity of activity- and pathology-dependent changes of KCC2 function

### KCC2 changes related to physiological patterns of neuronal activity

Whether physiological patterns of neuronal activity control developmental enhancement of KCC2 function remains controversial. At least three studies have provided data supporting the idea that chronic blockade of GABAergic (Ganguly et al., 2001; Leitch et al., 2005) or glycinergic (Shibata et al., 2004) inputs in developing neuronal networks inhibits the developmental increase in KCC2 functional expression. In contrast, other studies have not found significant changes in KCC2 functional expression after prolonged inhibition of GABAergic neurotransmission (Ludwig et al., 2003; Titz et al., 2003). The later conclusion does not exclude, however, that the activity might contribute to fine adjustments of the post-translational regulation (such as phosphorylation) and activation of plasmalemmal KCC2 function. Indeed, a critical role of the brain-derived neurotrophic factor (BDNF) and other trophic factors in the control of the developmental expression of KCC2 has been shown (Kelsch et al., 2001; Ludwig et al., 2011a,b). Given that neuronal activity regulates release of BDNF from both pre- and post-synaptic terminals (Kuczewski et al., 2008, 2010; Matsuda et al., 2009), it is plausible that BDNF and/or other trophic factors contribute to activity-dependent modulation of KCC2 in a context-dependent manner.

An important process in the maturation of glutamatergic networks is the specific form of plasticity resulting from co-incident pre- and post-synaptic spiking (Debanne and Poo, 2010). Similarly, spike-time-dependent plasticity has been shown to exist for GABAergic synapses (Woodin et al., 2003). Most intriguingly, within a sub-time domain, this new form of plasticity is dependent on changes of [Cl^−^]_i_. A suggested critical step in the mechanism is a rapid postsynaptic change in the efficiency of chloride extrusion by KCC2 (Fiumelli et al., 2005).

### Pathology-related changes of the KCC2 expression

Currently there is a large number of observations describing a strong decrease in KCC2 expression in different pathologies, including temporal lobe epilepsies (Palma et al., 2006; Huberfeld et al., 2007; Bragin et al., 2009; Barmashenko et al., 2011), focal cortical dysplasia (Munakata et al., 2007; Shimizu-Okabe et al., 2011; Talos et al., 2012), ischemia (Galeffi et al., 2004; Papp et al., 2008; Jaenisch et al., 2010), as well as pathologies associated with different types of axonal injury (Coull et al., 2003; Shulga et al., 2008; Boulenguez et al., 2010). Although the primary mechanisms initiating down-regulation of KCC2 during these pathologies are not known, the common point between them is a strong decrease of mRNA and protein expression levels. The exact mechanisms triggering inactivation of the KCC2 function are not yet well understood. Several groups have shown that epileptic-like neuronal activity itself could serve as triggering event. For instance, Nardou et al. (2009) showed suppression of the KCC2 function after induction of the epileptiform-like activity in the entire immature (P5-P7) rat hippocampal preparation. Rivera et al. (2004) found out that induction of epileptic-like activity to acute hippocampal slices rapidly (30–60 min) reduced expression of KCC2 in the CA1 region of the hippocampus and suggested a critical role of the glutamate and TrkB receptors in the processes. These results, however, were updated recently by Puskarjov et al. (2012) who showed that the half–life of plasma membrane KCC2 under control conditions might be significantly longer than previously reported. The difference between the two publications may be mainly attributed to differences in slice preparation. In the work by Puskarjov and collaborators, great care was taken to minimize damage of slices during preparation. In addition, they found that the mechanism for activity-dependent degradation of plasma membrane KCC2 requires Ca^2+^-dependent activation of the protease calpain. To achieve similar levels of KCC2 down-regulation as Rivera et al. (2004), the authors had to incubate slices with a higher concentration of NMDA (100 μM) and for a longer time (4 h). At these doses, NMDA triggers several neurotoxic signaling cascades (Hardingham et al., 2002; Ivanov et al., 2006), reviewed by Medina (2007) and Hardingham and Bading (2010). A rapid NMDA receptor-dependent KCC2 inactivation was also reported in two other studies performed on cultured hippocampal neurons (Lee et al., 2011; Pellegrino et al., 2011).

Many studies reporting a global pathology-dependent down-regulation of KCC2 expression mentioned also no-change or up-regulation of KCC2 in a sub-population of neurons surviving excitotoxic or ischemic insults. For example, Conti et al. (2011) demonstrated an up-regulation of KCC2 cotransporter in human peritumoral epileptic cortex (Conti et al., 2011). Huberfeld et al. (2007) have shown robust KCC2 staining in some subicular pyramidal cells in human temporal lobe epilepsy (TLE) brain sections. Jaenisch et al. (2010) have found a high level of KCC2 expression in infarct core neurons surviving a focal ischemic insult. Papp et al. (2008) found a delayed pattern of down-regulation in interneurons in the same model. Consistent with these data, Pellegrino et al. (2011) showed that all neurons surviving excitotoxic treatment with NMDA restored their normal Cl^−^ extrusion ability and increased their expression of KCC2.

While robust pathology-like activation of a neuronal network may lead primarily to inactivation of KCC2, short activity episodes might induce functional up-regulation. For instance, Khirug et al. (2010) found that a single seizure episode *in vivo*, as well as brief seizure-like activity *in vitro* in neonates, induced a potent increase in KCC2 plasma membrane expression (with unchanged total KCC2 expression) that correlated with enhancement in the efficacy of GABAergic inhibition. The up-regulated KCC2 activity was reported also in several other studies (Khirug et al., 2005; Banke and Gegelashvili, 2008; Chorin et al., 2011; Bos et al., 2013); however, it remains unclear how the described modulations of KCC2 activity are related to physiological or pathology conditions.

Since many of the discussed above reports show that restoration of the physiological level of Cl^−^ results in normal neuronal functioning, this suggests that targeting of the KCC2 and/or NKCC1 could be an efficient therapeutic strategy (reviewed by Kahle et al., 2008; Löscher et al., 2013). Regarding this, recent studies concentrated on high-throughput screening of the compounds capable to selectively inhibit the NKCC1 (Delpire et al., 2009, 2012) and activate KCC2 (Gagnon et al., 2013). Further optimization of the reported compounds as well as creation of new agents targeting specific signaling pathways upstream of KCC2 and/or NKCC1 will make in nearest future an important breakthrough for KCC2-related therapy.

## Cell-inherent levels of KCC2 control

### Transcriptional regulation of KCC2 expression

Most mature CNS neurons exhibit high levels of KCC2 expression, while low expression is detected in immature neurons (reviewed in Blaesse et al., 2009; Chamma et al., 2012). KCC2 mRNA levels are increased with neuronal maturation, KCC2 protein expression shows concordance with the mRNA level (Lu et al., 1999; Li et al., 2002; Stein et al., 2004). RNA interference approaches, such as anti-sense oligonucleotides (Rivera et al., 1999) or specific siRNAs (Bortone and Polleux, 2009; Pellegrino et al., 2011; Gauvain et al., 2011; Succol et al., 2012), lead to a reduction of KCC2 protein and a resulting loss of KCC2 activity. Thus, the control at the mRNA level is an efficient way to regulate the long-term activity of KCC2.

Selective decrease in KCC2 mRNA has been observed during neurological disorders (Palma et al., 2006; Huberfeld et al., 2007), after sustained interictal-like activity (Rivera et al., 2004), and after axonal injury (Nabekura et al., 2002; Toyoda et al., 2003; Shulga et al., 2008, 2009). Activity-dependent decreases in KCC2 mRNA might therefore be an important factor contributing to the down-regulation of KCC2 activity in certain pathological conditions. At least 10 putative transcription factor binding sites have been identified in the *SLC12A5* gene encoding KCC2 promoter and proximal intron-1 regions (Uvarov et al., 2006; Uvarov, 2010). These sites are highly conserved elements among promoter regions of other mammalian KCC2 genes. Transcription factor early growth response 4 (Egr4) has been identified as a potent regulator of KCC2 expression (Uvarov et al., 2006), which mediates BDNF-dependent KCC2 transcription in immature neurons (Ludwig et al., 2011b). Another regulatory element in the KCC2 promoter is the E-box, that binds the ubiquitously expressed upstream stimulating factors USF1 and 2, thus contributing KCC2 up-regulation in the developing cortex (Markkanen et al., 2008). In addition, two neuron-restrictive silencing elements (NRSE) were found in *SLC12A5*; however, the data on whether they are involved in the developmental up-regulation of KCC2 are controversial (Karadsheh and Delpire, 2001; Uvarov et al., 2005; Yeo et al., 2009). The role of other putative transcription factor binding elements in the KCC2 promoter remains obscure. One important task will be to characterize in more detail the regulatory elements controlling *SLC12A5* transcription.

### KCC2 assembly

Numerous studies suggest that, by analogy with other types of transporters and Cl^−^ channels, the CCC family members function as oligomers (McKee et al., 2000; Moore-Hoon and Turner, 2000; Casula et al., 2001; De Jong et al., 2003; Starremans et al., 2003; Simard et al., 2004; Blaesse et al., 2006; Parvin et al., 2007; Simard et al., 2007; Pedersen et al., 2008; Casula et al., 2009; Warmuth et al., 2009). With regard to KCC2, the ability of this molecule to form molecular complexes corresponding to mono-, di-, tri-, and tetrameric complexes was first demonstrated by Blaesse et al. (2006). The authors showed also a clear age-dependence of the oligomerization: the immature brains were characterized by higher content of the monomers, whereas adult tissue produced mostly oligomeric forms of the KCC2. An additional set of data demonstrating the ability of KCC2 to form dimers *in vivo* comes from studies of the distribution (Markkanen et al., 2013) and interaction (Uvarov et al., 2009) between KCC2a and KCC2b isoforms (Uvarov et al., 2007). Using the native perfluorooctanoate (PFO)-PAGE system, as well as a co-immunoprecipitation assay, Uvarov et al. (2009) have shown that KCC2a and KCC2b isoforms form homo- and heterodimers *in vivo* and in heterologous HEK-293 expression system. Despite multiple evidence describing oligomerization of KCC2, the functional context of this protein complex remains unclear. Is it critical for translocation to plasma membrane? What is the number of subunits that form the ion-transport unit? Is oligomerization involved in KCC2 surface expression and/or internalization? These and other questions remain to be answered and should be a subject for future studies.

**Figure 2:**
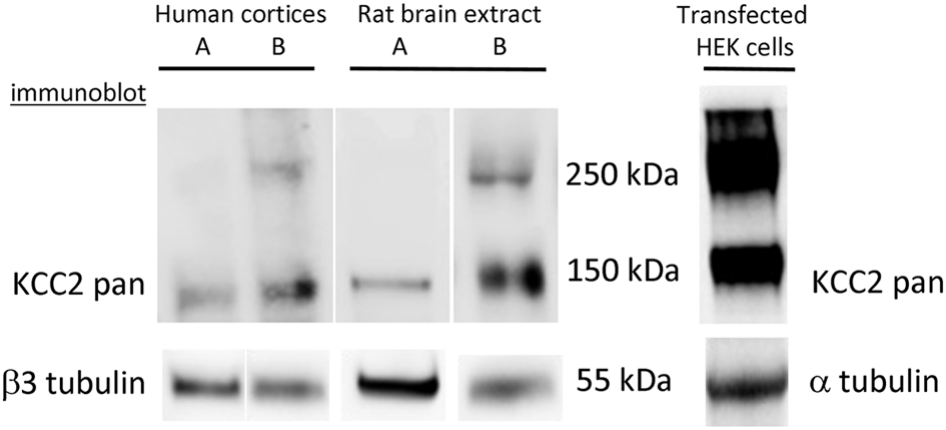
**Complexity of interpretation of KCC2 oligomerization**. Formation of KCC2 dimer-like complexes depends on the protein extraction procedure and is different for endogenous and over-expressed KCC2. KCC2 extraction using a rapid procedure (<1 min) either from human cortices or rat brains generates a single band of ~140 kDa. The extracts prepared from similar brain samples using the same solutions but with a longer overall time of extraction (> 4 min) always give at least two bands corresponding to monomer (140 kDa) and dimer (280–300 kDa) of KCC2. The extraction of eGFP-KCC2 over-expressed in HEK-293 cells resulted in the appearance of two bands independently on the procedure of extraction. Similar two-band expression profile of KCC2 was obtained after over-expression of different KCC2 forms (with or without additional tags) into different cell lines (PC-12 and Neuro2a).

By reviewing results of the KCC2 oligomerization studies, we have to be aware of some technical problems that one may encounter during analysis of the KCC2 using Western blot. Due to yet unidentified reasons, KCC2 is prone to forming SDS-resistant high molecular aggregates during standard protein extraction and solubilization procedures that are normally used for denaturation and dissociation of protein complexes (Gallagher, 2012). By performing Western blot analysis of the KCC2, we have found that in addition to the age-dependent changes in KCC2 oligomeric/monomeric ratio described in the above study (Blaesse et al., 2006), the ability to form oligomers strongly depends on the experimental procedure and is different for endogenously expressed KCC2 and for KCC2 overexpressed in heterologous systems (Figure 2). A very rapid protein extraction reduces the formation of the KCC2 oligomeric complexes under SDS-denaturating conditions, whereas long extraction (more than 4 min) increases the probability to get dimers. Interestingly, once the KCC2 dimer complexes are formed, we could not dissociate them to monomeric structure using boiling with SDS or in combination with high concentration (8 M) of urea at high temperature, a treatment that works efficiently to disrupt such strong molecular complexes as NMDA receptors-associated postsynaptic densities (Krapivinsky et al., 2003, 2004).

### Surface expression, turnover

Plasma membrane insertion, surface stabilization, and internalization of the molecule constitute one more important mechanism of KCC2 regulation. Numerous studies have found that pathological-like patterns of neuronal activity initiate rapid withdrawal of the KCC2 from plasma membrane that leads to a reduction of KCC2-mediated ion-transport (Rivera et al., 2004; Lee et al., 2007, 2010, 2011; Wake et al., 2007; Puskarjov et al., 2012; Chamma et al., 2013). Using an elegant approach of visualizing KCC2 internalization harboring an extracellular tag, Zhao and colleagues have shown a critical role of the cytoplasmic C- and not N-termini for KCC2 trafficking (Zhao et al., 2008). The authors also localized in the KCC2 C-terminus a novel non-canonical di-leucine motif (_657_LLXXEE_662_) that seems to be essential for the constitutive internalization of the transporter and for binding to the clathrin-binding adaptor protein-2 complex. Several phosphorylation sites on the C-terminus have been implicated in mechanisms controlling KCC2 turnover (see below “phosphorylation” chapter for details and Figure 1).

The internalization of overexpressed KCC2 is controlled by the clathrin-mediated endocytosis pathway in HEK-cells (Zhao et al., 2008) and can be prevented by an inhibitor of the clathrin pathway in cultured neuronal cells (Lee et al., 2011). However, no systematic work was performed to characterize the recycling mechanism involved or the signaling pathways controlling the KCC2 membrane insertion. A recent study (Puskarjov et al., 2012) launched the discussion on the timing of the KCC2 turnover in neuronal membrane. Previous work (Rivera et al., 2004), which was performed using a biotinylation approach and hippocampal slices prepared using a slice chopper (i.e., resulting in a significant number of damaged cells), suggested that half of the KCC2 molecules are renewed in neuronal plasma membrane within 30–40 min. Using a different experimental approach and slices prepared using a vibratome (i.e., with a lower rate of neuron damage), Puskarjov et al. (2012) estimated the half time of KCC2 turnover to be much longer (several hours or even days). On the other hand, a study performed using cultured hippocampal neurons showed that 45-min treatment of neurons with dynasore, an inhibitor of the endocytosis, produced twofold increase of the surface expression of the KCC2 (Lee et al., 2010), indicating the existence of a relatively rapid constitutive KCC2 endocytosis in this model. A further evidence supporting the high rate of the KCC2 turnover was obtained in a heterologous expression system (HEK-293 cells) where over-expressed KCC2 showed a half-time turnover rate close to 10 min (Lee et al., 2007; Zhao et al., 2008). Thus, the timing and mechanisms controlling KCC2 surface expression remain to be fully elucidated.

### Regulation of the intrinsic KCC2 activity

The structural elements of KCC2's ion-transport unit are not known. Unlike other members of the KCC family (KCC1, KCC3, and KCC4), which become active only after osmotic stress (reviewed by Flatman, 2008; Blaesse et al., 2009), KCC2 is able to extrude Cl^−^ in isotonic conditions. The over-expression of this transporter in heterologous expression systems (Payne, 1997; Williams and Payne, 2004) or neuronal cells (Chudotvorova et al., 2005; Lee et al., 2005; Pellegrino et al., 2011; Acton et al., 2012) produces a clearly detectable ion flux that could be rapidly (within few seconds) and reversibly inhibited using high concentrations of furosemide, a sulfamoylbenzoic acid “loop” diuretics (Payne, 1997; Williams and Payne, 2004; Friedel et al., 2013). These data suggest that, when expressed in the plasma membrane, KCC2 operates as constitutively active K^+^-Cl^−^ transporter.

Several groups have identified in the KCC2 molecule different regions and amino acid residues that regulate the ion-transport activity. Hartmann et al. (2010) found that mutation of four evolutionarily conserved cysteine residues in the large extracellular loop of KCC2 (C287, C302, C322, C331, Figure 1) inhibited ^86^Rb^+^ flux mediated by the transporter without affecting its surface expression. Interestingly, mutation of the corresponding cysteines in KCC4, which is the closest to KCC2 member of the *SLC12* family, did not affect the ion transport. These data suggest a distinct organization of ion-transport mechanism in KCC2 and KCC4, thus further enhancing exclusiveness of the KCC2. An important role in KCC2-mediated ion transport plays also C568, located inside the 10th putative transmembrane domain (Reynolds et al., 2008). Mutation of this residue (C568A) makes KCC2 inactive, but it remains unclear whether C568A mutation inhibits intrinsic activity or affects membrane targeting of the molecule. A study performed by Horn et al. (2010), described a decreased interaction of the KCC2_C568A_ mutant with cytoskeleton-associated 4.1N protein, and suggested that the C568A mutation causes changes in the tertiary structure of the molecule. Additional analysis is necessary to clarify the mechanism of how the C568A mutation silences KCC2 activity.

As illustrated in Figure 1, the N- and C- terminal domains of KCC2 and other members of KCC subfamily, are both located intracellularly. Several sets of data suggest that both domains can regulate intrinsic activity of the transporter. Thus, by expression in *Xenopus* oocytes of different KCC1 constructs with truncated intracellular regions, Casula et al. (2001) found that deletion of either N- or C- termini abolished ion transport activity of KCC1 without affecting transporter expression at the oocyte surface. Consistent with this finding, Li et al. (2007) showed that truncation of the N-terminus of KCC2 inhibited its ion-transport activity and did not affect the surface expression of the protein (Figure 1). A further evidence highlighting the importance of the KCC C-terminus in controlling the intrinsic property of ion transport was found in a study by Rinehart et al. (2009). Authors showed that phosphorylation of KCC3 at two newly identified sites (T991 and T1048) strongly inhibited the ion-transport activity of the transporter without modifying its surface expression. Age-dependent changes of the phosphorylation status of equivalent threonine sites in the KCC2 molecule (T906 and T1007) were reported in the same study; however it remains unclear whether phosphorylation of the KCC2 at these residues modifies its intrinsic activity or surface expression.

The leucine 675 (L675) identified by Döding et al. (2012) is one more residue critically involved in KCC2 activity: its mutation to alanine (L675A) strongly reduced ^86^Rb^+^ flux mediated by the KCC2 without affecting the surface expression of the transporter in mammalian cell lines.

It had been known for a long time that KCC2 could be constitutively active in isotonic conditions in HEK293 cells (Payne, 1997), CHO cells (Strange et al., 2000) and in *Xenopus* oocytes (Strange et al., 2000; Song et al., 2002; Gagnon et al., 2006a). In contrast to KCC2, several studies revealed no significant cotransporter activity of KCC4 under isotonic conditions, but found that KCC4 could be strongly (about 20-fold) activated by hypotonic swelling (Mercado et al., 2000; Song et al., 2002). To identify molecular mechanisms underlining this difference, Mercado and coauthors used Xenopus oocytes and multiple KCC2-KCC4 chimeric constructs, and found a C-terminal region unique to KCC2 (ISO-domain, amino acids 1021–1035) that is both necessary and sufficient for the isotonic KCC2 activity (Mercado et al., 2006). A recent study confirmed important role of ISO domain by illustrating in transfected cultured hippocampal neurons that KCC2 mutant with deleted ISO domain showed reduced ion transport activity, whereas insertion of the ISO domain into KCC4 transporter, which is inactive under isotonic conditions, rendered it fully operational (Acton et al., 2012). Of notice, by using different KCC2-KCC4 chimera constructs overexpressed in Xenopus oocytes, Bergeron et al. (2006) obtained almost identical to Mercado et al. (2006) results showing that the deletion of distal KCC2 region strongly reduced KCC2 activity under isotonic conditions, whereas insertion of this domain to KCC4 potently activated transporter. However, after analysis of many other KCC2 and KCC4 mutants authors were not convinced that the ISO segment is “a prerequisite for K^+^-Cl^−^ cotransport to occur under isotonic conditions.” Clearly, a characterization of the mechanism of ISO domain action is one more challenge in the KCC2 field.

Taken together, the above data suggest that both N- and C-terminal domains of KCC might be directly involved in the control of the ion-transport unit; however, the mechanisms of their regulatory actions are not known. An important task for future studies will be to answer these questions by analysis of the three-dimensional structure of KCC proteins.

## Signaling pathways that control KCC2 activity

**Figure 3:**
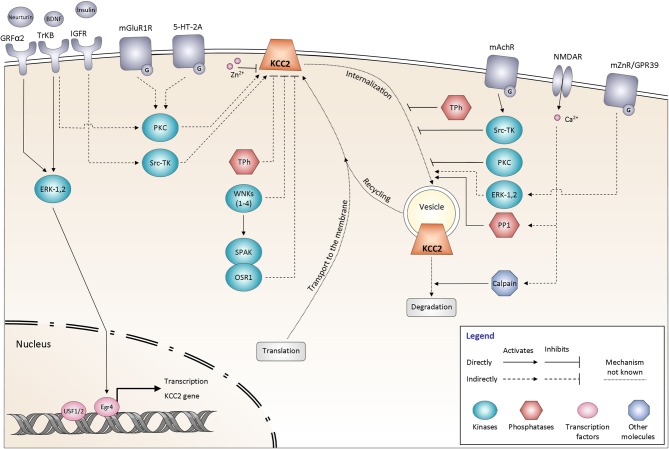
**Signaling pathways controlling KCC2 function**. The regulation of KCC2 activity is mediated by many proteins including kinases and phosphatases. It affects either the steady state protein expression at the plasma membrane or the KCC2 protein recycling. All the different pathways are explained and discussed in the main text. The schematic drawings of KCC2 as well as other membrane molecules do not reflect their oligomeric structure. GRFα2, GDNF family receptor α2; BDNF, Brain-derived neurotrophic factor; TrKB, Tropomyosin receptor kinase B; Insulin, Insulin-like growth factor 1 (IGF-1); IGFR, Insulin-like growth factor 1 receptor; mGluR1, Group I metabotropic glutamate receptor; 5-HT-2A, 5-hydroxytryptamine (5-HT) type 2A receptor; mAChR, Muscarinic acetylcholine receptor; NMDAR, N-methyl-D-aspartate receptor; mZnR, Metabotropic zinc-sensing receptor (mZnR); GPR39, G-protein-coupled receptor (GPR39); ERK-1,2, Extracellular signal-regulated kinases 1, 2; PKC, Protein kinase C; Src-TK, cytosolic Scr tyrosine kinase; WNKs1–4, with-no-lysine [K] kinase 1–4; SPAK, Ste20p-related proline/alanine-rich kinase; OSR1, oxidative stress-responsive kinase -1; Tph, Tyrosine phosphatase; PP1, protein phosphatase 1; Egr4, Early growth response transcription factor 4; USF 1/2, Upstream stimulating factor 1, 2.

Many signaling molecules have been implicated in the control of KCC2 function (Figure 3). Some of these molecules and pathways have been explored in detail, whereas many others remain to be characterized. Below, we overview these pathways based on their major regulatory functions.

### BDNF and other trophic factors

At least three trophic (growth) factors have been identified as efficient modulators of KCC2 functional expression: BDNF, Insulin-Like Growth Factor 1 (IGF-1), and Neurturin (Kelsch et al., 2001; Ludwig et al., 2011a; Shulga et al., 2012). Among them, the regulatory effects of BDNF on KCC2 are the most thoroughly characterized.

First evidence for the regulatory action of BDNF in developing neurons was presented by Aguado et al. (2003), who showed that *in vivo* transgenic over-expression of BDNF strongly increases KCC2 mRNA. Later the deletion of the receptor TrkB was shown to strongly decrease KCC2 mRNA (Carmona et al., 2006). Whether these effects are observed at the protein expression levels have so far not been reported in the literature. Consistent with this study, the application of BDNF to immature hippocampal cultures significantly increases the expression of KCC2 protein through Egr4-dependent KCC2 transcription (Ludwig et al., 2011b). Interestingly, in more mature cultures BDNF produced a decrease in KCC2 expression, thus revealing that the action of BDNF depends on the maturational state of neurons (see for review (Ferrini and De Koninck, 2013). So far, there is no evidence describing the regulatory action of the BDNF on the post-translational level at early stages of neuronal development.

In contrast to immature neurons, the exposure of adult neurons to BDNF *in vivo* and *in vitro* decreases KCC2 expression and function. BDNF decreases KCC2 mRNA levels (Rivera et al., 2002; Shulga et al., 2008), total KCC2 protein expression (Rivera et al., 2004), as well as tyrosine-phosphorylated (Wake et al., 2007) and membrane-inserted (Boulenguez et al., 2010) fractions of KCC2. Consistent with these findings, over-expression of BDNF *in vivo* produced a depolarizing shift of the GABA reversal potential in dorsal spinal cord lamina I (L1) neurons, presumably reflecting a decrease in KCC2 function (Coull et al., 2003, 2005).

Therefore, in mature neurons, application of exogenous BDNF induces a down-regulation of the KCC2 function. Endogenously released BDNF may therefore be a good candidate to mediate the activity-dependent down-regulation of KCC2. In support of this hypothesis, Rivera et al. (2004) showed that scavenging of endogenous BDNF, or inhibition of signaling downstream from BDNF TrkB receptors, effectively prevented activity-dependent reduction of KCC2 mRNA and protein. Several other studies have also reported a critical role of BDNF in the change of the inhibitory strength of GABA following axonal injury (Coull et al., 2005; Shulga et al., 2008; Boulenguez et al., 2010; Zhang et al., 2013). Intriguingly, BDNF application on neurons after injury, which are characterized by a decreased level of KCC2 expression, produced effects opposite to those observed in naive tissue, namely up-regulation of KCC2 surface expression (Shulga et al., 2008; Boulenguez et al., 2010). One plausible explanation is that injured neurons acquire properties of immature ones in order to launch a repair program (Kahle et al., 2008; Shulga et al., 2008, 2012).

Both during development (Westerholz et al., 2013) and in axotomized tissue (Shulga et al., 2009; Shulga and Rivera, 2013), the regulatory action of BDNF is tightly related to the thyroid hormone metabolism. The fact that thyroxine can bi-directionally regulate the expression of KCC2 in the same manner as BDNF would indicate that this effect could be mediated through BDNF signaling (Friauf et al., 2008; Shulga et al., 2009). Interestingly, the effect of thyroxin on KCC2 appears to be insensitive to BDNF signaling blockage indicating yet another pathway for regulation of KCC2 expression.

### Phosphorylation

It has been long time appreciated that K^+^-Cl^−^ cotransport in non-excitable cells can be potently regulated by phosphorylation (Russell, 2000; Blaesse et al., 2009). Extrapolating these data to neuronal K^+^-Cl^−^ transport, several studies have made an attempt to characterize the role of protein phosphorylation in the functioning of the KCC2 (Kahle et al., 2013). Large number of putative KCC2 phosphorylation sites were shown to contribute into the control of transporter activity and/or its surface expression (Figure 1). Numerous studies have highlighted also different phosphorylation-related signaling pathways contributing to the control of KCC2 activity (Figure 3). Below we review the recent evidence and make an attempt to classify them with regard to the phosphorylation type (serine/threonine and tyrosine) and kinases implicated into the post-translational regulation of the KCC2. We emphasize, in particular, the discrepancies between different works and highlight unsolved questions.

#### Serine-threonine phosphorylation

To date, serine 940 (S940), localized in the cytoplasmic C-terminal domain of KCC2, is the only known residue whose phosphorylation enhances KCC2 activity. This residue has been identified using *in vitro* screening of the KCC2-derived peptides directly phosphorylated by protein kinase C (PKC) (Lee et al., 2007). Dephosphorylation of S940 leads to inactivation of KCC2-mediated ion transport and increases endocytosis of the transporter. A high level of S940 phosphorylation is seen under resting conditions in heterologous expression systems and cultured hippocampal neurons, as revealed using an antibody raised against the phosphorylated form of KCC2 S940 (Lee et al., 2011). Thus, the PKC-dependent phosphorylation of S940 in the C-terminus of KCC2 is critical for maintenance of the KCC2 activity under physiological conditions. The mechanisms controlling S940 are not identified yet. At least three studies have described the PKC dependent regulation of KCC2 during physiologically-relevant processes including: (i) activity-dependent attenuation of the KCC2 function in immature cultured neuronal cells (Fiumelli et al., 2005), (ii) tonic activation of the KCC2 by group I metabotropic glutamate receptors (mGluR1s) (Banke and Gegelashvili, 2008), and (iii) activation of the KCC2 via serotonin type 2A receptors (Bos et al., 2013). However, it remains unclear whether PKC-dependent regulation of KCC2 activity in the above-mentioned studies, was direct or via some intermediate signaling molecules. Treatment of cultured hippocampal neurons with glutamate provoked a rapid dephosphorylation of S940 residue that was NMDA receptor and protein phosphatase-1 dependent (Lee et al., 2011). An important task would be to determine whether the described signaling pathway contributes to decrease of the KCC2 activity following neurological pathologies. Lee et al. (2007) also identified another serine residue (S728) whose mutation to alanine (S728A, dephosphorylated-like form) produced a constitutive increase of the KCC2 activity. Unfortunately, the authors did not study how S728 phosphorylation modifies the surface expression of the transporter and did not identify the pathway controlling this phosphorylation site.

Rinehart et al. (2009) identified two threonine residues conserved in other KCC members (T906 and T1007 on the isoform b of rat KCC2 (accession number NP_599190)) whose phosphorylation strongly inhibited the transporter activity, whereas dephosphorylation rendered transporters constitutively active. T906 is partially phosphorylated in neonatal mouse brain and dephosphorylated in adult brain, suggesting a potential contribution of T906/T1007 dephosphorylation to functional KCC2 up-regulation during development. However, a systematic analysis of KCC2 phosphorylation at these sites in discrete anatomic regions during brain development is required. Engineering KCC2 T906A (or E)/T1007A (or E) double knock-in mice would be valuable in this regard.

The WNK (with-no-lysine [K]) family of serine-threonine kinases comprises four members (WNK1, WNK2, WNK3, and WNK4) that together with SPAK, a Ste20p-related proline/alanine-rich kinase (Johnston et al., 2000) and OSR1, an oxidative stress-responsive kinase -1 (Tamari et al., 1999) form a powerful signaling cascade involved in control of swelling-induced regulation of CCC family protein members (see for review Delpire and Gagnon, 2008; Kahle et al., 2010). All these kinases are expressed also in the CNS, but their functional role in neurons is not known. Importantly, the activity of the WNK-SPAK pathway is regulated by extracellular osmolality and intracellular Cl^−^ concentration (Zagórska et al., 2007; Thastrup et al., 2012). The 4 members of WNK family have been shown to effectively inhibit the activity of the KCC2 cotransporter exogenously overexpressed in neurons (Inoue et al., 2012) or in *Xenopus* oocytes (Kahle et al., 2005; De Los Heros et al., 2006; Gagnon et al., 2006b; Rinehart et al., 2011). Taken together, the above evidence indicates the importance of detailed evaluation of the WNK-SPAK/OSR1 phosphorylation cascade in control of the KCC2 function.

Toward this goal, the role of KCC2 T906 and T1007 phosphorylation during brain development was recently studied by Inoue et al. (2012) using an elegant *in utero* electroporation approach and over-expression of KCC2 mutants mimicking hyper- and de-phosphorylated forms of transporter (T906E + T1007E and T906A + T1007A, respectively) in rat cortex. Inoue et al. (2012) suggested that the phosphorylation of these residues in immature brains is maintained by taurine acting through the WNK-SPAK/OSR1 phosphorylation cascade. The proposed scheme was based, however, exclusively on evidence obtained using application of exogenous taurine and exogenous over-expression of wild type and mutant KCC2 and WNK1 kinase constructs.

An important future task would be to verify the proposed hypothesis using models involving silencing of the endogenous molecules (WNK and SPAK), and to biochemically demonstrate how inhibition of WNK-SPAK alters KCC2 phosphorylation. Very recently de Los Heros et al. revealed that the WNK-regulated SPAK/OSR1 kinases directly phosphorylate KCC2 at T1007 (site 2), but not at T906 (site 1), and inhibition of WNK-SPAK signaling with a chemical inhibitor *activates* K-Cl cotransporter activity in cells by decreasing T1007 > T906 inhibitory phosphorylation in ES cells (De Los Heros et al., 2014). SPAK/OSR1 is also necessary for KCC2 T1007 but not T906 phosphorylation in ES cells. This is of high interest, given efforts aimed at finding chemical activators of KCC2 to restore ionotropic inhibition. Perhaps a novel way of activating KCC2 in certain cellular contexts would be to oppose the action of inhibitory kinases, such as WNK/SPAK (Kahle et al., 2013). While there are no classic knockout mouse models available for WNK1 or OSR1 due to their requirement for embryogenesis (Xie et al., 2013), exploring the roles of SPAK (and other WNKs, like WNK3) in KCC2 regulation *in vivo* should be possible using knockout mice (Yang et al., 2010; Mederle et al., 2013) along with other transgenic mice which express SPAK harboring a point mutation that renders them unable to be activated by WNK kinases (Rafiqi et al., 2010).

#### Tyrosine phosphorylation

KCC2 phosphorylation at tyrosine residues (TP, tyrosine phosphorylation) was shown in several studies (Stein et al., 2004; Wake et al., 2007; Watanabe et al., 2009; Lee et al., 2010), however its importance for KCC2 functioning remains obscure. While some studies showed the existence of the KCC2 TP under physiological conditions in rodent's cortex (Stein et al., 2004) and primary hippocampal cultures (Wake et al., 2007; Watanabe et al., 2009), others did not observe the basal TP under control conditions in cultured hippocampal neurons, although strong TP was observed after cultures treated with phosphatase inhibitors (Lee et al., 2010). Wake et al. (2007) clearly showed that neuronal network activity, oxidative stress, and treatment with BDNF all lead to a strong *decrease* of KCC2 TP. In contrast, Lee et al. (2010) observed a robust *increase* of KCC2 TP in brain slices obtained from rats after pilocarpine-induced status epilepticus, as well as in primary neuronal cultures treated with carbachol - an agonist of acetylcholine receptors. Finally, by comparing the influence of different experimental conditions on the KCC2 TP level, E_GABA_, and distribution of the KCC2 protein levels, Wake et al. (2007) and Watanabe et al. (2009) concluded that “*direct tyrosine phosphorylation of KCC2 results in membrane clusters and functional transport activity”* (Watanabe et al., 2009). However, based on the comparison of KCC2 TP levels, surface expression of the biotinylated KCC2 fraction, and total KCC2 expression, Lee et al. (2010) provided an opposite conclusion stating that KCC2 tyrosine phosphorylation promotes KCC2 internalization and lysosomal degradation.

Many explanations for these discrepancies are possible; however the most reasonable one seems to be that the treatments utilized to activate/inhibit TP in the mentioned studies were different. All employed treatments (H_2_O_2_, BDNF, 0 Mg^2+^, carbachol, pilocarpine, blockers of kinases and phosphatases) are able to modify the activity of different signaling pathways, including differential modulation of tyrosine kinases and phosphatases, as well as serine/threonine kinases, which itself can produce dual modulation of the KCC2 when acting through S940 or T906/T1007 (see above). Clearly, additional studies are required to clarify the question.

So far, two amino acid residues (Y903 and Y1087) were identified on KCC2 as potential carriers of tyrosine phosphorylation signal (Lee et al., 2010). Authors found that mutations of these residues to phenylalanine (Y903/1087F), which mimic dephosphorylated state, almost fully abolished the tyrosine phosphorylation state of the KCC2 protein exogenously overexpressed in HEK-293 cells. Using biotinylation assays, the authors showed that the Y903/1087F KCC2 mutant strongly increases the surface expression of KCC2, providing an additional argument for the TP-dependent internalization and degradation of KCC2. While the Y903 residue was characterized only recently, the Y1087 amino acid has been a subject of several studies that have resulted in contradictory conclusions. The Y1087 residue was first highlighted on a widely used KCC2 scheme presented by Payne et al. (1996) as the tyrosine residue conserved through evolution in all members of KCC and NKCC family transporters. Strange et al. (2000) showed that mutation of this residue to aspartate (Y1087D, mimicking a hyperphosphorylated state) fully abolished activity of the KCC2 transporter, whereas Y1087F mutation did not modify the KCC2-mediated ion transport. The authors, however, did not observe the change in the surface expression of the mutants in the *Xenopus* oocyte expression system, and they also could not find any experimental evidence to support the importance of tyrosine phosphorylation in the control of KCC2 activity. A full inactivity of KCC2 construct carrying Y1087D mutation and overexpressed in brain-derived GT1-7 cell line was also reported by Watanabe et al. (2009). Consistent with observation by Strange et al. (2000), authors did not find any changes in the surface expression of the Y1087D mutant compared to the wild type KCC2 (biotinylation assay). Interestingly, in their study, Watanabe et al. (2009) considered the Y1087D mutant of KCC2 as non-phosphorylated mutant. This was surprising as aspartate has totally different structure as compared to the tyrosine. Usually, to mimic the non-phosphorylated state of tyrosine researches use phenylalanine, an amino acid identical to tyrosine, with the exception of the absence of the phosphorylatable hydroxyl group (see Anthis et al., 2009 for detailed discussion on the strategy of tyrosine residues replacement). Given that Strange et al. (2000) found a strong difference in the activity of Y1087F and Y1087D mutants, the Y1087D mutation should be rather considered as phosphorylation-like mutation. Unfortunately, in their work Watanabe et al. (2009) did not compare the action of Y1087D with alternative Y1087F mutant. The inactivity of Y1087D mutant was confirmed several other times in other heterologous systems and cultured neurons (Chudotvorova et al., 2005; Akerman and Cline, 2006; Pellegrino et al., 2011). The above mentioned studies, however, used the Y1087D mutation as an experimental tool and did not analyse the mechanisms leading to the inactivation of KCC2 activity.

Thus, there is a good agreement between all existing studies that Y1087D mutation, that potentially mimics the phosphorylated-like state of the residue, inactivates KCC2 overexpressed in oocytes (Strange et al., 2000), mammalian cell lines (Watanabe et al., 2009) and neurons (Chudotvorova et al., 2005; Akerman and Cline, 2006; Pellegrino et al., 2011). In opposite, the mutation Y1087F, mimicking de-phosphorylated-like state of KCC2 residue, does not affect KCC2 transport in oocytes (Strange et al., 2000) and potentiates it in HEK-293 cells (Lee et al., 2010). Taken together, these data strongly suggest that Y1087 phosphorylation reduces KCC2 activity, whereas its de-phosphorylation potentiates the transporter. Due to discrepancies in the results characterizing the surface expression of Y1087D mutants, the mechanism controlling KCC2 activity (change in the surface expression vs. change of the intrinsic activity) remains unclear.

What is the relationship between Y903/Y1087 phosphorylation and endogenous KCC2 tyrosine phosphorylation? The Y903/Y1087 residues were detected in HEK-293 expression system as unique phosphorylation residues whose mutation fully abolished exogenous KCC2 phosphorylation. These data do not exclude an existence of additional residues accessible for phosphorylation by tyrosine kinases in native neuronal environment. The phosphorylation of these other residues might produce opposite effects than mutations in Y903/1087. Therefore, one cannot exclude differential effects of TP on endogenous KCC2 and on KCC2 mutated at one or two tyrosine amino acids.

Clearly, more scrupulous quantitative analysis is required to answer the following questions: (i) what is the basal level of KCC2 tyrosine phosphorylation in different *in vitro* preparations (acute slices, organotypic slices, primary neuronal cultures) compared to maximal phosphorylation level achieved in presence of the activators of the TP and inhibitors of the phosphatases, (ii) what is the basal level of the KCC2 TP *in vivo* (compared to *in vitro* models), (iii) how many tyrosine residues could be phosphorylated in native neuronal environment, (iv) what are the functional consequences (ion-transport activity, surface expression, protein degradation) of the phosphorylation/dephosphorylation of the KCC2 at distinct tyrosine residues?

Finally, numerous studies provided evidence of the KCC2 control via at least two distinct TP-dependent pathways involving cytosolic c-Scr kinase (Kelsch et al., 2001) and BDNF-dependent TrkB receptor tyrosine kinase (Rivera et al., 2002, 2004; Coull et al., 2005; Boulenguez et al., 2010). Each of these kinases might regulate large number of signaling cascades. An important task would be to uncover the exact regulatory pathways controlling KCC2 activity.

### ZN^2+^-dependent control

Interesting data from groups of Hershfinkel and Aizenman (Hershfinkel et al., 2009; Chorin et al., 2011; Saadi et al., 2012) describe bi-directional Zn^2+^-dependent control of KCC2 (Figure 3). On one hand, intracellular Zn^2+^ in micromolar concentrations rapidly inhibits KCC2 activity (Hershfinkel et al., 2009). Although the mechanism of Zn^2+^ action is not clear, the finding indicates existence of a mechanism allowing inhibition of the intrinsic activity of KCC2. On the other hand, extracellular Zn^2+^ released from mossy fiber terminals, strongly activates KCC2 by increasing its surface expression (Chorin et al., 2011). Putatively, metabotropic zinc-sensing receptor (mZnR) is encoded by orphan Gq-coupled receptor (GPR39) and is coupled to phospholipase C (PLC)/extracellular-signal-regulated kinases (ERKs) pathway; the silencing of GPR39 or inhibition of PLC or ERKs abolished the Zn^2+^-dependent activation of KCC2 (Chorin et al., 2011). Consistent with a high rate of KCC2 turnover (Rivera et al., 2004), the authors have suggested that the increased surface expression of KCC2 is due to the inhibition of transporter internalization and/or degradation (Chorin et al., 2011). However, given recent evidence (see below chapter “Surface expression, turnover” and Puskarjov et al., 2012), one cannot exclude that Zn^2+^ potentiates the plasmalemmal insertion of the KCC2. The Zn^2+^-dependent control of KCC2 activity is one more subject in the KCC2 field requiring detailed analysis.

### Protein-protein interaction

**Figure 4:**
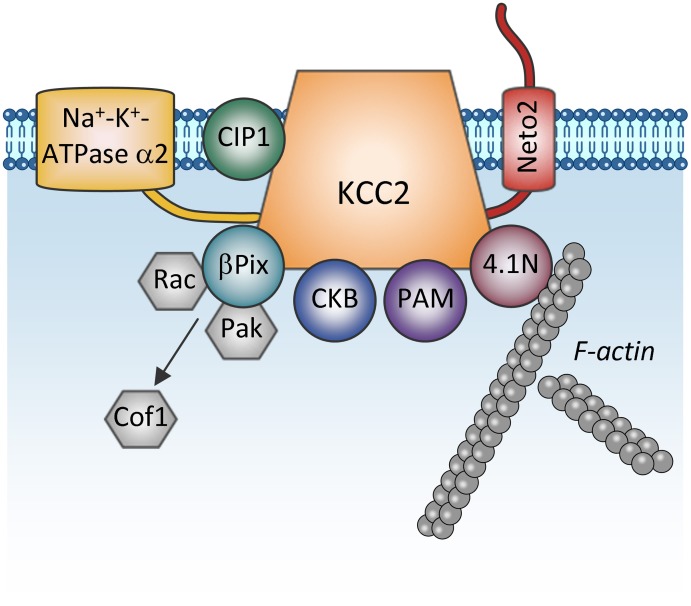
**Proteins directly interacting with KCC2**. The scheme shows membrane and cytoplasmic proteins that have been found to directly interact with KCC2. It is currently unclear whether these proteins form one big KCC2 containing complex or whether they all are combined in multiple distinct KCC2 containing pools (e.g., plasmalemmal vs. sub-membrane vesicular pools). Na^+^-K^+^-ATPase α2, subunit α2 of the Na^+^-K^+^-ATPase pump; CIP1, the cation-chloride cotransporter interacting protein 1; Neto2, neuropilin and tolloid like-2; β Pix, beta isoform of the Rac/Cdc42 guanine nucleotide exchange factor; Rac, small GTPase of the Rho family; Pak, p21-activated serine/threonine-protein kinase kinase; Cof1, cofilin-1; CKB: brain-type creatine kinase; PAM, protein associated with Myc; 4.1N, cytoskeleton-associated protein.

One more regulatory mechanism of KCC2 activity is through its direct interaction with other proteins (Figure 4). The first such interaction was described by Inoue et al. (2004) showing that brain-type creatine kinase (CKB) interacts with KCC2 and activates the transporter in HEK-293 cells (Inoue et al., 2004, 2006). Ikeda et al. (2004) found an intriguing interaction between the α2 subunit of the Na-K-ATPase pump and KCC2 that may stand for the breathing malfunction found in mice lacking the Na-K-ATPaseα2 subunit (Atp1a2^−/−^). These mice display decreased functional activity of KCC2 and have abnormal spontaneous respiratory rhythm activity, similarly to the KCC2^−/−^ mice. KCC2 interacts also with other unrelated proteins such as the protein associated with Myc (PAM) via its RCC1 (Regulator of Chromatin Condensation) domain, resulting in the functional regulation of KCC2 activity in HEK-293 cells (Garbarini and Delpire, 2008), or with the cation-chloride cotransporter interacting protein 1 (CIP1) (Wenz et al., 2009), but the functional significance of this latter link in neurons remains unknown.

Furthermore, the cytoskeleton-associated protein 4.1N interacts with the cytoplasmic carboxy-terminus of KCC2 and is involved in dendritic spine formation (Li et al., 2007) and the plasticity of AMPA receptors (Gauvain et al., 2011). Interrupting the KCC2:4.1N interactions enhanced lateral diffusion of KCC2 away from excitatory synapses (Chamma et al., 2013). These results indicate a role for 4.1N in the local stabilization of KCC2 at the plasma membrane. Although lateral KCC2 diffusion was enhanced by NMDA receptor activity, the pathophysiological relevance of this is unknown.

Another molecule interacting with KCC2 is the single-pass transmembrane protein neuropilin and tolloid like-2 (Neto2) (Ivakine et al., 2013). Neto2 is required to maintain the normal abundance of KCC2 and specifically associates with the active oligomeric form of the transporter. Loss of the Neto2:KCC2 interaction reduced KCC2-mediated Cl^−^ extrusion, resulting in decreased synaptic inhibition in hippocampal neurons. Presently, the activity-dependent pathways controlling KCC2 via interaction with Neto2 are unknown, but consistent with Neto2 being part of a neuronal scaffolding platform (Tang et al., 2012), other studies will likely characterize the role of the KCC2:Neto2 interaction in neuronal plasticity or during excitotoxic events that affect the scaffold protein organization.

A recent finding describes a direct interaction between KCC2 and the beta isoform of the Rac/Cdc42 guanine nucleotide exchange factor (betaPIX). The synergetic action of KCC2 and betaPIX leads to decreased cofilin phosphorylation and change of glutamatergic synapse properties (Ludwig et al., 2013) but the importance of this interaction for activity-dependent signal transfer needs to be determined.

### Lipid rafts

At least two studies have suggested an important role of the lipid rafts in the control of KCC2 activity (Hartmann et al., 2009; Watanabe et al., 2009). Lipid rafts are specialized membrane microdomains, enriched in glycosphingolipids and glycoproteins, serving as organising centers for the assembly of signaling molecules and control of their activity (for recent review see Lingwood and Simons, 2010). Both, Hartmann et al. (2009) and Watanabe et al. (2009) illustrated that in neurons approximately 50% of KCC2 is associated with lipid raft markers, whereas remaining transporter molecules are linked to non-rafts markers. However, author's conclusions on the importance of the function of the rafts-like organization for KCC2 were opposite: Hartmann et al. (2009) claimed that, when in rafts, KCC2 is in an inactive form, whereas Watanabe et al. (2009) were convinced that functional KCC2 forms rafts-like clusters. These contradictory assumptions, however, were made based on results obtained using different experimental paradigms, by employing different and wide-range acting pharmacological agents, and by exploring KCC2 mutants whose properties require more detailed analysis. Therefore, after careful and detailed critical analysis of both studies we propose that KCC2 might be membrane raft-associated protein, but additional experimental works are required from independent groups to clarify the role of lipid rafts in KCC2 functioning.

In summary, KCC2 is an important transporter controlling neuronal ion homeostasis and involved in several neurological disorders. The activity of this transporter could be rapidly modulated via large number of signaling pathways. The majority of these pathways remain to be characterized. In the Box 1, we review the existing methods used to study KCC2 activity in an attempt to help investigators in designing experiments to answer the many fundamental questions regarding KCC2 functional regulation.

### Conflict of interest statement

The authors declare that the research was conducted in the absence of any commercial or financial relationships that could be construed as a potential conflict of interest.
